# A reassuring presence: An evaluation of Bradford District Hospice at Home service

**DOI:** 10.1186/1472-684X-7-9

**Published:** 2008-08-01

**Authors:** Beverley Lucas, Neil Small, Peter Greasley, Andrew Daley

**Affiliations:** 1 Senior Lecturer Pharmacy Education, School of Life Sciences, University of Bradford, Richmond Road, Bradford, BD7 1DP, UK; 2 Professor of Health Research, School of Health Studies, University of Bradford, 25 Trinity Road, Bradford, BD5 0BB, UK; 3 Lecturer, School of Health Studies, University of Bradford, 25 Trinity Road, Bradford, BD5 0BB, UK; 4 Consultant in Palliative Medicine at Bradford Marie Curie Hospice and Bradford Teaching Hospitals NHS Foundation Trust, Duckworth Lane, Bradford, BD9 6RJ, UK

## Abstract

**Background:**

Within the United Kingdom, a developing role for primary care services in cancer and palliative care has resulted in an increase in palliative home care teams. The provision of professional care in the home setting seeks to provide necessary services and enhanced choice for patients whose preference is to die at home.

A mismatch between patient preference for home death and the actual number of people who died at home was identified within Bradford, the locality of this study. In response to this mismatch, and reflecting the policy environment of wishing to enhance community service provision, the four Primary Care Trusts (PCTs) in the city sought to offer support to patients who wished to remain in their own homes through the final stages of a terminal illness. To offer this support they set up a dedicated hospice at home team. This would provide services and support for patients in achieving a dignified, symptom free and peaceful death, allowing families to maximise time spent together. The aim of the study was to evaluate the Bradford hospice at home service from the perspective of carers, nurses and General Practitioners.

**Methods:**

Postal questionnaires were sent to carers (n = 289), district nurses (n = 508) and GP's (n = 444) using Bradford's hospice at home service. Resulting quantitative data was analysed using the Statical Package for Social Sciences (SPSS) and qualitative data was analysed using grounded theory techniques.

**Results:**

The data from carers, district nurses and GPs provide general support for the Bradford hospice at home service. Carers valued highly the opportunity to 'fulfil a promise' to the individual who wished to be cared for at home. District nurses and GPs cited the positive impact of access to specialist expertise. This was a 'reassuring presence' for primary healthcare teams and offered 'relief of carer anxiety' by providing prompt, accessible and sensitive care.

**Conclusion:**

Carers and health professionals welcomed the increased possibility of patients being cared for at home. The study identified the need to focus on improving skill levels of staff and on ensuring continuity of care.

## Background

The provision of professional care in the home setting has become an integral component of healthcare delivery in most western countries [1]. This mode of care is evident in UK palliative care and is consistent with an emphasis on the pivotal role of primary care services in cancer and palliative care [2], enhanced support for family carers [3] and a commitment to honour more patient choice [4]. The White Paper *Our health, our care, our say *[5] commits the National Health Service (NHS) to a shift in focus to provide integrated health and social care services in local communities, closer to people's homes.

There is an established body of evidence which identifies dying at home as the preferred choice of both the general public and primary healthcare professionals [6-9], although there is also a recognition that preferences may change during the course of an illness [10]. However, the patient's preferred place of death is not always achieved [11] and thus there is a failure to fulfil this aspect of people's end-of-life care wishes [12].

In recent years there has been an increase in the numbers of palliative home care teams and, in consequence, enhanced choice for some patients who wish to remain at home [13-15]. The effectiveness of home care interventions have been considered in a number of studies, each with different emphases.

• A review of UK palliative care literature identified some evidence of efficacy of home care when considered from the patient point of view [16].

• District nurses' working alongside hospice at home teams reported a favourable impact in enabling patients with advanced progressive disease to be cared for at home [15].

• Exley and Tyrer [17] found, in the main, positive responses from bereaved carers commenting on the end of life care from a hospice at home service.

• King et al [18] focused on a rapid-response service and assembled views from service providers and carers, again finding high levels of satisfaction.

• Grady and Travers [19] reported high levels of satisfaction with the rapid response service they evaluated as well as significant improvement in some areas of pain and symptom management.

• Grande et al [20] evaluated the impact on place of death of a hospital at home service. Their randomised controlled trial could not show that hospital at home allowed more patients to die at home (neither did the study refute this).

### Local context

An internally circulated local audit, undertaken in Bradford in 2000, identified a mismatch between patient preference for dying at home and actual place of death. Seventy percent of patients with cancer indicated that they would like to die at home whilst only 23% did so. Issues identified by patients and health professionals to explain this disparity included: a) carer fatigue, b) difficulties in providing nursing care within the home setting, c) lack of appropriate staff skills in palliative support, d) problems in ensuring continuity of care, e) lack of team-working between agencies, and f) overall lack of availability of staff. Since the 2000 audit the specialist palliative care services in Bradford and Airedale have recorded patients' preferences, identified how many achieved their choice and sought to clarify reasons why an expressed preference was not met [21].

**Figure 1:**
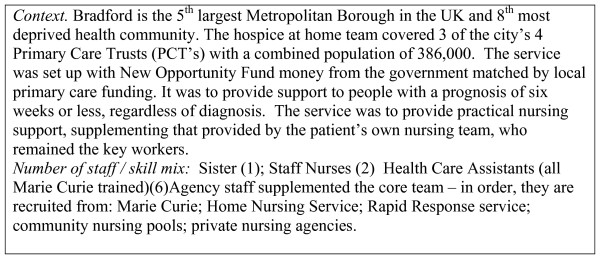
Description of the service at the outset of this study (2001).

In July 2001 a hospice at home service began in the city. (For details of the service see Fig 1). The service proposal that formed the basis for obtaining financial support both from the government's New Opportunities Fund and from Marie Curie Cancer Care included a commitment to seek a dignified symptom free and peaceful death, whilst allowing families to maximise time spent together.

We report results of an independent evaluation of Bradford hospice at home Service, addressing the following key questions:

• What were carers' perceptions of the value of the service?

• How did district nurses evaluate the contribution of the hospice at home team?

• How did GPs perceive the value of the support provided by the hospice at home team in facilitating patient choice to die at home?

## Methods

Process evaluation [22] offers an opportunity for evaluators to give information and assistance to service providers through feedback [23]. It is an approach that views relationships with practitioners as a priority [22]. Throughout this study the independent evaluators engaged with stakeholders to examine how the development of the hospice at home service impacted on care provision. The identification of areas of strength and issues for consideration informed service development so that the service would get nearer what was seen to be best practice by stakeholders.

The identified caregiver, district nurse and GP involved in the care of all patients referred to the service were eligible for inclusion in this study. Data was collected from the inception of the service in July 2001 to June 2006. The criteria for referral of patients to this City wide service met the Bradford District Continuing Care Criteria (2001) and the West Yorkshire Continuing Care Criteria (2002) at Level 6 (Continuing Care Funding).

A questionnaire, generated by the service delivery team and used within practice as part of clinical audit, was considered by the hospice at home steering group for inclusion in this evaluation. The questionnaire was proving to be acceptable to service providers and to carers. There appeared to be a satisfactory level of completion and an absence of critical comments about either the included content or about any omissions. The steering group decided that given that this tool worked in practice this should be considered as having constituted, in effect, an appropriate pilot for the evaluation study re the appropriateness and acceptability of the questionnaire. When the questionnaire was presented for ethical approval there were no revisions required. However the Ethics Committee did require additional safeguards to be added for the carer, specifically in the form of an informed consent form and a letter offering support for bereaved caregivers if required.

Exclusion criteria for this study were:

• When patients/carers changed their decision about taking up referrals resulting in no hospice at home input, e.g. they remained in hospital

• Patient had no identified carer (e.g. lived on their own)

• Patient transferred to alternative services, e.g. Marie Curie Service, nursing home or hospital

Of these three exclusion criteria, patient transfer appeared to be the most significant, followed by patient/carer changes of decision and the balance could be attributed to patients with no identified carer. However clinicians had not always recorded the criteria for exclusion and consequently this breakdown must be considered as an indicative rather than definitive finding.

Seven weeks after the death of a patient each identified main caregiver received a letter and informed consent form. If individual consent was given twelve weeks after the patients death they received a 15 item postal questionnaire. This sought carers' perceptions of:

* Quality of nursing

* Medical support

* Sense of dignity and respect

* Satisfaction with responsiveness of the hospice at home service

* Carer participation in the care package and emotional support.

* Place of death

The questionnaire included an invitation to add comments on any aspect of the health and social care services.

Following the death of a patient referred to the service and eligible for this study, a 12 item postal questionnaire was sent to the district nurse responsible for the care of the individual concerned. Questions were on the following themes:

* Setting up of care packages

* Shared approaches to care

* Communication with the hospice at home team

* Support offered to carers.

GP's of every eligible hospice at home patient who had died were sent a 3 item questionnaire to comment on perceptions of:

* Extent of patient and carer support

* Awareness of any patient or carer anxieties that were not addressed

* Management of death within the patients' home.

### Data analysis

Both quantitative and qualitative data were obtained from the questionnaires. The quantitative data were analysed using Statistical Package for Social Sciences (SPSS) frequency analysis.

The qualitative comments were typed verbatim and, given the modest allocation of space within the questionnaire for general comments, one aspect that surprised the team was the richness of free text feedback. In some examples, carers had appended extra typescript or hand written pages and others provided copious handwritten response both in the limited space allowed on the questionnaire and in the questionnaire margins. Many respondents clearly felt a need to expand on their experiences in areas not covered enough for them by the straightforward options of the questionnaire itself. There are many analytic procedures that can be used for qualitative data and this study adopted open and axial coding and memo-writing techniques drawn from the principles of grounded theory [24,25] to assist category generation. In practice, this necessitated the process of breaking down, examining and labelling data, then making connections between a category and sub-categories. To keep track of this process, written records of analysis were documented including summarising memos. Despite the laborious and time consuming implications of the decision to use this procedure this was considered worthwhile by the team because it would allow for the development of emergent rather than imposed categories from the data. This was necessary because this rich free text material suggested that issues were being engaged with and views expressed that moved beyond the pre-determined categories of the questionnaire. The team felt that this approach would also provide material to explore aspects of the data not used in this evaluation in subsequent papers, perhaps concentrating on specific understandings as to the nature of the care experience. Deciding upon open and axial coding and memo-writing also allowed us to generate in vivo codes that consisted of words and phrases used by participants themselves. These words and phrases included vivid imagery [26] such as 'fulfilling a promise' and 'reassuring presence' which we have used as key concepts in what follows.

The decision to use grounded theory techniques for data analysis within this study (rather than adoption of the grounded theory approach) links with a more general debate about technique versus method within grounded theory and with the importance of not claiming more in the terminology describing the procedures used than was actually carried out in practice [27]. These data analysis techniques are only one aspect of grounded theory and it was not the intention of the team to utilise a grounded theory approach (e.g. there was no theoretical sampling to saturation). Full application of grounded theory is unlikely to be used in small-scale evaluation, but the basic process of ordering codes into categories which are empirically related in the data as well as theoretical justified can be applied [22]. Codes were also independently analysed to examine fit.

### Ethical approval

Research governance approval was obtained from the Research and Development Unit Bradford South and West Primary Care Trust and ethical approval from Bradford Research Ethics Committee.

## Results

### Carer Questionnaire

During this study 1023 patients were referred to the hospice at home service. After applying the study exclusion criteria 453 carers were excluded. Of the 570 eligible for the study 289 (50.7%) of bereaved carers returned questionnaires.

#### Summary of results from yes or no questions in questionnaire to Carers: See Table 1

**Table 1:** Summary of results from yes or no questions in questionnaire to Carers

	Yes		No		Total
Question	N	*%*	N	*%*	

Q2. Could you have done with more nursing help?	57	*20*	230	*80*	287
Q3. Did you feel that the nurses knew enough about X condition and how to care for him/her?	262	*92*	23	*8*	285
Q5: Do you recall any difficulties the nurses experienced in obtaining medical support or medication for X?	51	*18*	231	*82*	282
Q6. Were agency staff used to support the care package?	211	*76*	65	*24*	276
Q7: Did the doctor from the Out of Hours Deputising Service visit at any time?	137	*49*	143	*51*	280
Q11. Did you have any difficulties contacting the Hospice at home Team?	21	*7*	265	*93*	286
Q13. Did X die at home?	250	*87*	39	*13*	289

In this article, we will focus on three key themes identified in carer data; quality of nursing care, medical support and, place of death.

#### 1) Quality of the nursing care

The first question to carers asked them to rate how they felt about the quality of nursing care in the last days of life on the following scale: 'very poor', 'poor', 'fair', 'good', 'very good'. Virtually all carers were positive about the quality of nursing care: 97% (n = 277) rated this as 'very good' (77%), or 'good (20%). Five carers rated it as 'very poor' or 'poor'.

The majority of carers 92% (n = 262) agreed that the nurses knew enough about the patients condition and how to care for him/her. Whilst carers were not asked to provide qualitative comments, a small number (3) wrote on the questionnaire about 'nurses helpfulness, understanding and patience'. However, 8% (n = 23) did not agree and free text comments (from 20) identified lack of 'understanding of condition' and lack of 'continuity of care'. As one carer commented:

It would have been more helpful to (patient) if more of the nurses had understood and known more about lymphodema and been able to help him with exercises and putting stockings on etc. [Comment number 111]

Problems identified relating to the continuity of care included this comment:

Continuity would have helped ..at first (they) did know how to move him without hurting him and what he needed. Some who didn't know him didn't understand the condition and what was happening therefore were not so careful with movement, etc. Also everything had to be explained every time. [Comment number 111]

Whilst 80% (n = 230) of carers felt that there was sufficient nursing help, 20% (n = 57) would have liked more. Qualitative comments (from 47) highlighted a belief that additional practical help with difficulties associated with patient immobility would have been welcome both in the form of more people being available, extra 'pairs of hands', and visits being more frequent :

Let me say initially that this service was a Godsend to me. Certain times when it was necessary to move (patient) more help would have been appreciated. [Comment number 304]

One particular problem relating to the quality of nursing care that was highlighted by carers was the use of agency staff. Seventy six per cent of carers (n = 211) identified that agency staff were used to support care delivery. There were a number of qualitative comments on this aspect of care (107) and a limited number of these (39) identified positive aspects of agency staff support such as 'friendly', 'good help in a trying situation'. In two cases agency staff was rated as 'excellent':

Agency staff were very caring and helped all they could to make X comfortable at home. [Comment number 279]

The majority of comments (68), however, were critical, commenting on a resulting 'variability' of care, including a perceived 'lack of confidence' in some agency staff abilities and in some cases 'lack of availability' of care staff.

All the time it was 24/7 – hardly ever the same staff -some were good – some not. I only had one agency girl who I wasn't keen with. She had the TV on all night, no ID. No uniform etc. I asked just for hospice girls. [Comment number 81]

Three were very poor, one bitched about her work saying she was packing it in. One wouldn't sit with [patient] and her husband came early for her (12.45 pm Sat) but claimed time till 1.00 am. One night sitter lectured me on God saying it was his will that [patient] should suffer.' [Comment number 89]

Three times they did not turn up. Once she rang in sick and we were told, but twice they just did not appear and one was on the night she died. [Comment number 122]

We will consider service attempts to address these serious problems with agency staff below. These attempts underline the process nature of this evaluation in that reported observations from the evaluation contributed to a realisation of the imperative to implement service changes.

#### 2) Medical Support

The majority of carers, eighty two percent (n = 231), reported no difficulties in obtaining medical support. The eighteen percent (n = 51) who experienced difficulties were invited to provide additional comments. There were 39 of these and they identified, most typically, 'lack of medical support' and 'lack of availability of drugs within the locality'.

The doctor on duty refused to visit or issue prescription, our son was in agony from 10 pm until 6.0.am. This was disgusting, unprofessional and cruel and was only slightly relieved by the care and concern shown by the nurses. [Comment number 104]

Insufficient morphine at chemists even though they confirmed enough by phone. [Comment number 100]

As with agency staff we will see below how observations such as these were acted upon prompting changes in service during the periods of this evaluation.

Forty nine per cent (n = 137) of carers had accessed medical support from the Out of Hours Deputising Service. In terms of frequency of access, the majority, 77% (n = 96), on one (n = 51) or two occasions (n = 45). Thirteen percent (n = 16) had three visits and four percent had either four visits (n = 5) or five (n = 5). There were two carers who had accessed the service on six and eight occasions respectively. Most people did not see the same deputising service doctor twice. There were a number of qualitative comments (36) that identified positive comments in relation to 'efficient caring service' and in the following example support under difficult circumstances:

All doctors were extremely supportive and worked hard to achieve the level of care in very difficult circumstances – [Patient] symptoms were very hard to treat. [Comment number 286]

Other responses (54) identified variability including perceptions of 'unacceptable delay in visits' and communication difficulties:

Before Mum was admitted to hospital we called the doctor which was Out of Hour's service – the call was 12.30. The doctor arrived at the following 9.30 – called ambulance – hospital 10.30. Not very happy with service (own doctors called on Mum every other day and were great). [Comment number 176]

Our own doctor visited when (patient) came home – this was during normal times. But through some lack of communication eight or nine hours elapsed before a doctor came to certify my husband's death. [Comment number 129]

### District Nurse Questionnaire

District Nurses returned 508 (89%) of questionnaires (no reminder questionnaires were sent).

In 95% (n = 463) of cases the district nurses identified that there had been a shared approach to care with the hospice at home team. Table 2 provides more detail of this relationship in practice. (Not all returned questionnaires were fully completed). There is evidence here that this shared approach is manifest in planning services and in the collaboration that continues throughout the delivery of care. But the provision of such care remains a source of considerable strain on the District Nurses, 38% (n 157) reported "undue strain" in supporting the patient to die at home.

**Table 2:** Summary of results from questionnaire to District Nurses

	Yes		No		Total
Question	N	*%*	N	*%*	

Setting up the Package					
Q1. Did you set up the initial package of care?	296	*58*	212	*42*	508
Q2. Were the hospice at home team involved in the initial planning of care?	399	*82*	86	*18*	485
Q3. Did hospice at home Team change input of care package?	222	*45*	267	*55*	489
Shared approach to care/Communication					
Q4. Do you feel that you contributed to the design of care?	486	*97*	17	*3*	503
Q5. Evidence of a shared approach?	463	*95*	26	*5*	489
Q6. Were you kept updated by hospice at home?	436	*90*	47	*10*	483
Q7. Was the care package delivered as you understood it would be?	453	*93*	35	*7*	488
Levels of support					
Q9. Was your perception that the family coped well with the level of support offered?	413	*88*	57	*12*	470
Q11. Could you have supported the patient to die at home without hospice at home?	102	*23*	333	*77*	435
Impact on district nurse workload					
Q12. Did supporting the patient at home cause undue strain on the District Nurse team?	157	*38*	253	*62*	410
Place of death					
Q10. Death at home?	404	*86*	67	*14*	471
Was the place of death the appropriate one?	450	*97*	14	*3*	464

#### Summary of results from questionnaire to District Nurses: See Table 2

A small number of comments reported the burden on caregivers, where 24 hour cover was problematic and carers were struggling to cope with meeting the patient's wishes.

[Patient] had needs during the night when the family found it most difficult to cope and on several consecutive occasions sitters did not attend. [Comment number 203]

It soon became apparent that family could not cope. [Comment number 237]

The family found the whole situation physically and mentally difficult. [Comment number 243]

Not a reflection of the support offered, but the carer would not have coped as she didn't really want her husband to stay at home but was trying to respect his wishes. [Comment number 371]

There were many comments added to the questionnaire thanking the hospice at home team for 'good work', 'expertise' and for being a 'reassuring presence' for other health professionals. There was general recognition that the service was crucial in terms of being able to support individuals in their preference for dying at home.

We would not have been able to nurse [patient] at home without input from hospice at home. Their input was vital, to the family, both day and night and to the district nurse team. Because of [patient] his young wife and two children, the hospice at home team gave them support, physical and emotional day and night. [Patient] was comforted by their presence in the house as was [patient's] parents. I would also like to thank you all for the help and support you gave me and my team, it was much appreciated. [Comment number 266]

[Patient] was in retention of urine – the GP or myself did not pick this up as the family stated (patients) pads were wet. The hospice at home nurses palliative care experience and knowledge was paramount in this particular incident and we were grateful to the input. The collaborative approach proved to be very positive. [Comment number 353]

Areas of concern identified included the importance of both availability of staff (including access to 24 hour/bank holiday cover) and concerns about appropriately educated and trained staff (with particular reference to agency health care assistants). These concerns were of importance because they could bring about a mismatch between patient and carer expectations and the actual service received.

Sometimes the service is let down by the lack of staff – families feel let down when they have been promised sitters and then there aren't any [Comment number 438].

Main carer very upset initially as patient unfortunately died the first night a sitter was arranged. Apparently the sitter informed the carer that patient had died without waking her when his condition deteriorated. Upset not to have been with him at the time of death [Comment number 106].

### General practitioner questionnaire

GPs returned 444 (78%) of distributed questionnaires (no reminder questionnaires were sent).

GP's comments underlined the benefit of the service in respecting the wishes of patients and/or carers for home to be the place of death.

#### Summary of results from questionnaire to General Practitioners: See Table 3

**Table 3:** Summary of results from questionnaire to General Practitioners

	Yes		No		Total
**Question**	N	*%*	N	*%*	

Q1. Overall did you feel that the patient and family were sufficiently supported?	427	*96*	17	*4*	444
Q2. Were you aware of any family/patient anxieties, which were not addressed?	38	*9*	401	*91*	439
Q3. Did you feel managing this death at home caused any additional concerns within the practice?	47	*11*	394	*89*	441

The general practitioner responses to their questionnaire revealed an overwhelmingly positive perception that the patient and family were sufficiently supported (96%: n = 427). Free text comments included:

Was accepted for treatment quickly and seen quickly – initiated promptly and were able to respect patients/family's wishes of dying at home. [Comment number 410]

The main thing was that (patient) wanted to die at home. Without the support of the team she would have needed to go into hospital or hospice care. [Comment number 205]

GP's also outlined the key importance of the service in terms of providing emotional support for patient and carers and support for the primary healthcare team.

(Patient) did not live long after her diagnosis but I know that her daughter and son felt supported by hospice at home and found her easier to manage. [Comment number 256]

(A strength was) knowing I had expert advice available if I needed it. [Comment number 313]

The importance of working together for better care featured in comments:

Obvious moral, medical and psychological support to the patient and family. Communication line was efficiently kept between GP/care people and patient and family. [Comment number 386]

Good teamwork between hospice at home, nurses and GPs [Comment number 442]

Enabled a very sick woman to come home from hospital and die comfortably at home with her family [Comment number 304].

Of the 4% (n = 17) who identified lack of sufficient support, GP's echoed carer responses in terms of problems with the 'sitting service' (particularly overnight and weekend) and three comments suggested communication between primary and secondary services could have been improved. These comments included:

We were told no staff available to offer patient support over weekend as waiting list – this resulted in patient's emergency admission to hospital. [Comment number 255]

The communication from secondary and tertiary care was poor. (This is not a criticism of hospice at home service, which was fine but not started soon enough). [Comment number 151]

Ninety one percent (n = 401) of GPs were not aware of any unaddressed family or patient anxieties. Of the comments received 9% (n = 38) of GPs remarked on the difficulties of caring, often in rapidly deteriorating circumstances, for individuals within the home;

Her son panicked at the time of death, although death was expected, and dialled 999 that led to a chain of undesirable events, which probably [patient] didn't want. [Comment number 266]

This man [patient] and his wife needed a little more help to face directly the issue of death. [Comment number 116]

Not a good death 'unexpected collapse' paramedics attempted to resuscitate. But mostly due to patients refusal to discuss how he saw things going when he became unwell towards the end. [Comment number 365]

### Death at home

The primary aim of establishing the hospice at home service was to support, with best quality care, patient choice to die at home. Here, reflecting the importance of this aspect of the service, we present further data from all three groups in the study specifically addressing this aspect of care.

Carer questionnaires reported that 87% (n = 250) of patients had died within the home. There were prolific carer comments (211) about the importance of 'patient choice', the 'reassuring presence' of the hospice at home service and the importance of 'fulfilling a promise' in terms of the individuals preferred place of death.

I couldn't have gone through the whole thing without them, it was my wife's desire to die at home and I'm so grateful for the support to enable her wish to be fulfilled. [Comment number 234]

He, I and our family, found this to be a very precious time. A time to say things he needed to say, get his affairs settled, his wishes made known and for myself and our family to give him our love [Comment number 80]

A key strength identified by carers was the importance of their active participation in cares, thereby, 'fulfilling a promise' to individuals who had expressed a preference to die at home. There were many general comments (117 recorded) on questionnaires thanking the team.

Mum passed in the best way possible; at home, pain free with all her family around her. I will be forever grateful to the district nurses and hospice at home team for allowing this and for helping her to die with dignity. [Comment number 16]

My husband wished to have his care up to his death at home, it was also my wish. His passing was dignified and peaceful with his family at his side in his own bed in accordance with his wishes – wholly appropriate in our case. [Comment number 121]

The overwhelming experience of District Nurses was that in hospice at home cases place of death was appropriate even where the place of death was not the home.

He [patient] was admitted to ward – he asked to go into hospital rather than at home, it was his wish. [Comment number 467]

(Patient) was admitted to hospice for symptom control – her death was sudden. [Comment number 507]

District Nurses also suggested that it would not have been possible to support the client within the home without the hospice at home service. For example:

The hospice at home team is a must if we are to nurse the dying at home. The district nurses can only give limited time on visits due to their caseloads. The hospice at home team can provide the care and support the patient and family require whilst going through this difficult situation. [Comment number 348]

Eighty nine per cent of GPs (n = 394) reported no additional concerns in terms of managing death within the home, and welcomed the support from the hospice team.

I think we feel adequately supported by good access to the palliative care team. Overall we have found this an excellent service for our patients. [Comment number 443]

Eleven percent (n = 47) reported that managing death at home had caused additional concerns. These related to practical matters around the challenge of symptom control, repeated visits and out of hour's medical cover:

This caused a lot of strain in the practice due to the amount of time we needed to spend there, the difficulty in controlling the symptoms and the awfulness of the whole situation. [Comment number 251]

We had concerns re out-of-hours doctor (previous concerns with other patients). Practice doctors provided additional support at weekends. [Comment number 434]

## Discussion

We will organise our discussion of study results in two sections. First we will comment on methodological and interpretive aspects of the study and then we will consider key features of the hospice at home service as identified by our respondents.

### Methodological and interpretive issues

#### 1) The challenge of positive findings

Replies that are overwhelmingly positive can be difficult to deconstruct in ways that are useful for service providers to translate into guides for service improvements [28]. Further, asking about satisfaction with care in palliative care is especially challenging. In the absence of an easy outcome measure of cure or remission and in the likelihood that care is provided in the context of worsening symptoms and more complex care demands, it is a challenge to conceptualise clear expectations about what services might even consist of let alone realistically achieve. Satisfaction needs to be related to both expectations and aspirations [29]. These challenges are likely to be evident in all three of our respondent groups but to be particularly pronounced in our carer respondents. Whilst carers could only compare hospice at home support with either their expectations or any service they had received before the introduction of hospice at home to their situation the district nurses and GPs had a broader range of comparative experiences to draw on. Offering three different but contemporaneous perspectives on the Bradford hospice at home service is a particular strength of this study.

Wilkinson et al [30] have commented on difficulties exploring consumer opinion on, and satisfaction with, specialist models of palliative care. These difficulties further underline the methodological problems of data collection within a palliative care environment. Even so, they suggest that consumer perspectives of the quality of communication, access to care, problem areas and valued aspects of care are important in terms of the development of future models and as such should not be overlooked by funding agencies or managers of palliative care services.

#### 2) Using findings to improve care

Our use of qualitative comments is designed to move beyond the reassurance that questionnaires on satisfaction with care can generate to illustrate the care experience in the more nuanced way that qualitative analysis permits. Relative to the proportion of negative comments offered overall in the study we have presented in our results section a disproportionate number of critical qualitative comments. These comments highlight the points of tension in service delivery and give a focus for considering interventions to improve the overall quality of care. Two specific areas can be explored further, the shortcomings of agency staff and, in the next section, the impact of the service on rates of death at home.

Our use of both questionnaire and qualitative data does facilitate the identification of shortcomings in terms of the depth of expertise available to the team, when staff, outside of the core team were utilised problems increased. This has significant resource and training implications not just for increasing the quality of end-of-life care in the home but also for broadening the range of this service and of similar services elsewhere.

The experience of having agency staff involved in care in the home is the reason for a considerable number of the critical comments elicited in this study. A significant number of these criticisms appear to be of major concern; our quotes presented above illustrate how distressing some of these avoidable experiences were. It is essential to address how these shortcomings might be overcome.

In the early days of the hospice at home service in Bradford there were few core staff employed and a reliance on agency staff resulted. Over time this situation has changed, as has the degree of scrutiny exercised by the hospice at home team over those agency staff who continue to be involved. Crucial to the shift from agency towards core staff was a successful funding bid, in 2003, to expand the team and the Marie Curie nursing budget. This allowed a reduction in the use of agency staff. There had also been a more systematic process of recording problems with particular agency staff and feeding these back to the appropriate agency via the PCT Contracts Manager. Educational and training opportunities were made available to specific agency staff. Further, as the team became better supported financially those agencies with whom problems were most evident were dropped. Contract specifications and minimum service requirements were strengthened within the contracting process for those agencies that remain involved.

As well as these attempts to address the problems of agency staff this evaluation has encouraged other quality of care improvements to be made; a) in enforcing the need for and acceptability of setting a standard for maximum waiting time before visit to palliative care patients by an out-of-hours doctor. This has largely been adhered to by the new 'Out of Hours' provider, b) demonstrating the need for a robust system of providing injectable palliative care drugs out of hours (including opioids). Again, this is now largely in place locally, with all doctors' cars carrying an agreed list of drugs.

#### 3) The complexity of planning for death at home

One of the main aims of the introduction of a hospice at home service in Bradford was to increase the home death rate. In the period of this evaluation this was not achieved. The relationship between service innovation and home death rates is a complex one. Gomes and Higginson [31] undertook a systematic review of 58 studies and identified a complex mix of 17 factors that impacted on place of death and preferences for death at home being met. These factors included patient specific factors, including their functional status, preferences and patterns of available family support; patterns of service provision including the intensity of home care. They concluded that future policies and clinical practice should focus on ways of empowering families and public education, as well as intensifying home care, risk assessment and training for practitioners in end of life care.

Even within an overall policy context supportive of home care, services are vulnerable to changes within primary healthcare teams that arise for other agendas relevant to this area of practice, for example changes in GPs out of hour's contracts [32] and access to drug dispensing [33].

### Key features of the service

#### 1) Sensitivity to carer support needs

A key strength identified by the majority of carers was the importance of their active participation in care, thereby, 'fulfilling a promise' to individuals who had expressed a preference to die at home. Whilst Cantwell et al [34] found the main predictive factor of a home death was the agreement between the patient desiring a home death and the caregiver supporting a home death a relatively small minority of carers in our study reported the high level of stress involved in caring for someone until they died at home, even with the support of the hospice at home team. It is important to recognise that patient preference may not always be the choice for carers. A disparity like this presents a challenge for families and also for healthcare professionals that must be acknowledged and addressed.

Despite there being carers who reported such a high level of stress the 'reassuring presence' of the Bradford hospice at home service provided much needed support for the majority. Healthcare professionals also reported on the value of the service in building carer trust and facilitating patient choice. Factors associated with continuity of care and the importance of effective team-working are paramount if patients, carers and healthcare professionals are to have overall confidence in a home care service.

#### 2) Team-working: continuity of care

Whilst team work is considered a central component of palliative care [35] findings from other hospice at home team evaluations have suggested problematic areas in communication between district nurses and hospice at home teams [36,15]. In terms of providing continuity of care, results of one multi-centre evaluation suggested the advantages of all components of care being offered by one service [14]. Carers of patients referred to another hospice at home service have also reported the importance of accessibility of primary care professionals, which becomes more acute within the home with lay carers often on their own looking after individuals while experiencing the anxiety and strain surrounding impending death [37]. Thomas et al [38], exploring change in planned and actual location of death, identified carer anxiety and lack of confidence as important determinants as to why place of death is often different from patients preferred option.

Within our study, carers, district nurses and GP's cited examples of working together for better care within the home with co-ordination viewed as a priority. In a small number of cases, where difficulties with providing 24-hour care were encountered, e.g. sitters not turning up or perceptions of inappropriate night cover, this impacted negatively on carers and healthcare professionals confidence in the service.

#### 3) Problems in prognosis

The considerable majority of patients seen by this hospice at home team had cancer and it may be that challenges in prognosis contribute to the exclusion of people with other conditions. Anecdotally, we identified that in some cases GPs were referring people with non-cancer diagnosis and claiming a prognosis of six weeks or less in order to give their patients access to the enhanced services hospice at home could offer. In effect the inexact science of prognosis is being used in two ways – one to exclude some people and one to include them in this sought after service even when GP's may not have an evidence based judgement to reassure all parties that the six week prognosis was realistic. In effect the six week rule was manipulated to maximise care for individual patients. Subsequent to the period considered in this evaluation there has been further encouragement to increase the scope of the hospice at home service to offer palliative care services to patients with life limiting conditions other than cancer who expressed a preference for home death.

### Study limitations

There are limitations in this study arising from its focus on one health community. The study provides an insight into the views of caregivers, district nurses and GP's. It does not include direct patient views of home care provision nor does it monitor the quality of palliative care within the home using measures of, for example, pain and other symptom relief [39]. The anonymous data does not allow matching of carer, district nurse and GP opinion. There may be a disproportionate tendency of those who are satisfied with services to reply to questionnaires. The requirements imposed on the study by the research ethics committee included sending an information letter and informed consent form seven weeks after the death of the patient. If consent was given, the carer received a questionnaire five weeks later. The time scale was chosen to balance the likelihood of accurate recollection with the emotional and practical impact of the bereavement. It may have had an adverse impact on carer recruitment.

The study was carried out within a service context that saw the hospice at home service experience high staff turnover, considerable pressure of work and the absence of effective administrative support in its early years. This real world context is not untypical of much health and social care research and the shortcomings, like less than ideal response rates and incomplete data sets should be viewed in this context.

However there are also advantages in this close involvement of the research process with service delivery. Much health service research is essentially evaluative and the alliance between researcher and practitioner is predicated on an assumption that the orientation of research is to help service improvement, to have an impact, to make a difference [40]. What then can become a sense of shared endeavor helps to ensure continued enthusiasm for the research. It also offers a route for the rapid transmission of research findings to service providers and their managers. Close involvement of research also provides a spur for improvements in the quality of routine data collection. Much of the data that is collected by practitioners is often not of sufficient quality to be used to provide or underpin research findings and can even be of little value for audit or service planning.

## Conclusion

The study provides an independent evaluation of the Bradford hospice at home service and reports positive responses from patients informal carers', district nurses and general practitioners. However while there was only a small number of times that care was not seen positively when this did occur the level of distress at such a crucial point in carers lives can be considerable. In end-of-life care if one does not get things right first time it is not likely that a less than optimal situation can be remedied. Even where 97% of carers rate care as good or very good for the small numbers who reported poor or very poor care it was for 100% of their relatives end-of-life experience. This puts considerable pressure on end-of-life care to get it right every time. It is a pressure that needs to be acknowledged by commissioners, trainers and managers.

One of the main aims of the introduction of a hospice at home service in Bradford was to increase the home death rate. It is regrettable that this aim was not fulfilled. Given the range of factors that impact on home death rates [20,31] it was ambitious to think the introduction of this service would realise this aim.

It does emerge from all parties that the sensitive and reliable care of high quality provided by the hospice at home service offered a reassuring presence and it was this that defined the quality of the end-of-life experience. While a hospice at home service does have a responsibility to engage with its broader public health impact and so needs to consider the impact it has on the proportion of those people who choose and then experience their death at home we have observed above that factors that bear upon this are wider than the remit of hospice at home. What is within its direct remit is the process of care delivery and it is this and not, more narrowly, just the place of death that determines the success of the service for those who received it.

## Competing interests

The authors declare that they have no competing interests.

## Authors' contributions

NS: Conceived the study, acquired the funding, and participated in design, co-ordination of the research and analysis of data. BL: Undertook data collection and managed, liaison with the service, undertook analysis and contributed towards the full report to the funders. AD: Contributed towards the design and links with the clinical service. PG: Analysed quantitative data.  All authors contributed to the final manuscript.

## Pre-publication history

The pre-publication history for this paper can be accessed here:



## References

[B1] Miller K-L, McKeever P, Coyte P (2003). Recruitment issues in healthcare research: the situation in home care. Health and Social Care in the Community.

[B2] Department of Health (2000). The NHS Cancer Plan.

[B3] National Institute for Clinical Excellence (2004). Guidance on Cancer Services: Improving Supportive and Palliative Care for Adults with Cancer.

[B4] Richards M National Clinical Director: Cancer Reform Strategy.

[B5] Department of Health (2006). Our health, our care, our say: a new direction for community services.

[B6] Dunlop RJ, Davies RJ, Hockley JM (1989). Preferred versus actual place of death: a hospital palliative care support team experience. Palliative Medicine.

[B7] Townsend J, Frank AO, Fermont D, Dyer S, Karran O, Walgrove A, Piper M (1990). Terminal cancer care and patients' preference for place of death: a prospective study. BMJ.

[B8] Cartwright A (1991). Balance of care for the dying between hospitals and the community: perceptions of general practitioners, hospital consultants, community nurses and relatives. Br J Gen Pract.

[B9] Grande GE, Addington-Hall JM, Todd CJ (1998). Place of death and access to home care services: are certain patient groups at a disadvantage?. Soc Sci Med.

[B10] Hinton J (1994). Can home care maintain an acceptable quality of life for patients with terminal cancer and their relatives?. Palliative Medicine.

[B11] Franks PJ, Salisbury C, Bosanquet N, Wilkinson EK, Kite S, Naysmith A, Higginson J (2000). The level of need for palliative care: a systematic review of the literature. Palliative Medicine.

[B12] Foreman LM, Hunt RW, Luke CG, Roder DM (2006). Factors predicative of preferred place of death in the general population of South Australia. Palliative Medicine.

[B13] Boyd KJ (1994). Hospice care in the United Kingdom. Ann Acad Med Singapore.

[B14] Clark D, Ferguson C, Nelson C (2000). Macmillan Carers Schemes in England: results of a multicentre evaluation. Palliative Medicine.

[B15] Sullivan KA, McLaughlin D, Hasson F (2005). Exploring district nurses' experience of a hospice at home service. International Journal of Palliative Nursing.

[B16] Ingleton C, Payne S, Nolan M, Carey I (2003). Respite in palliative care: a review and discussion of the literature. Palliative Medicine.

[B17] Exley C, Tyrer F (2005). Bereaved carers' views of a hospice at home service. International Journal of Palliative Nursing.

[B18] King G, Mackenzie J, Smith H, Clark D (2000). Dying at home: evaluation of a hospice rapid-response service. International Journal of Palliative Nursing.

[B19] Grady A, Travers E (2003). Hospice at home 2: evaluating a crisis intervention service. International Journal of Palliative Nursing.

[B20] Grande GE, Todd CJ, Barclay SIG, Farquhar MC (1999). Does Hospital at home for palliative care facilitate death at home? Randomised controlled trial. British Medical Journal.

[B21] Daley A, Sinclair K (2006). Recording and auditing preferred place of death. Palliative Medicine.

[B22] Hall I, Hall D (2004). Evaluation and social research.

[B23] Øvretveit J (1998). Evaluating health interventions.

[B24] Glaser BG, Strauss AL (1967). The discovery of grounded theory.

[B25] Strauss AL, Corbin J (1998). Basics of qualitative research; techniques and procedures for developing grounded theory.

[B26] Glaser BG (1978). Theoretical sensitivity.

[B27] Kennedy TJT, Lingard LA (2006). Making sense of grounded theory in medical education. Medical Education.

[B28] Small N, Green J, Spink J, Forster A, Lowson K, Young J (2007). The patient experience of community hospital – the process of care as a determinate of satisfaction. Journal of Evaluation in Clinical Practice.

[B29] Fakhoury WKH (1998). Satisfaction with palliative care: what should we be aware of?. International Journal of Nursing Studies.

[B30] Wilkinson EK, Salisbury C, Bosanquet N, Franks PJ, Kite S, Lorentzon M, Naysmith A (1999). Patient and carer preference for, and satisfaction with, specialist models of palliative care: a systematic literature review. Palliative Medicine.

[B31] Gomes B, Higginson IJ (2006). Factors influencing death at home in terminally ill patients with cancer: systematic review. BMJ.

[B32] King N, Thomas K, Bell D (2003). An out-of-hours protocol for community palliative care: practitioners' perspectives. International Journal of Palliative Nursing.

[B33] (2004). National Forum for Hospice at Home: Submission to Health Select Committee. http://www.hospiceathome.org.uk/.

[B34] Cantwell P, Turco S, Brenneis C, Hanson J, Neumann CM, Bruera E (2000). Predictors of home death in palliative care cancer patients. Journal of Palliative care.

[B35] Junger S, Pestinger M, Elsner F, Krumm N, Radbruch L (2007). Criteria for successful multiprofessional cooperation in palliative care teams. Palliative Medicine.

[B36] Melvin J (2003). Gold standard palliative care in the community. Primary Health Care.

[B37] Grande GE, Farquhar MC, Barclay SIG, Todd CJ (2004). Valued aspects of primary palliative care: content analysis of bereaved carers' descriptions. British Journal of General Practice.

[B38] Thomas C, Morris SM, Gatrell AC, McIllmurray MB (2001). Place of death in Morecambe Bay; patterns and preferences for place of final care and death among terminally ill cancer patients and their carers. Morecambe Bay Medical Journal 3.

[B39] Mercadante S, Fulfaro F, Casuccio (2000). The impact of home palliative care on symptoms in advanced cancer patients. Supportive Care in Cancer.

[B40] Robson C (2002). Real world research: a resource for social scientists and practitioner-researchers.

